# Use of online knowledge base in primary health care and correlation to health care quality: an observational study

**DOI:** 10.1186/s12911-020-01313-9

**Published:** 2020-11-16

**Authors:** Christian Gerdesköld, Eva Toth-Pal, Inger Wårdh, Gunnar H. Nilsson, Anna Nager

**Affiliations:** 1 Division of Family Medicine and Primary Care, Department of Neurobiology, Care Sciences and Society, Karolinska Institutet, Alfred Nobels Allé 23 D2, 141 83 Stockholm, Sweden; 2 Department of Dental Medicine, Academic Centre of Geriatric Dentistry, Karolinska Institutet, Stockholm, Sweden; 3 Academic Primary Health Care Centre, Region Stockholm, Sweden; 4 Medibas, Bonnier Healthcare Sweden, Stockholm, Sweden

**Keywords:** Clinical decision support, Information systems, Knowledge bases, Registries, Primary care

## Abstract

**Background:**

Evidence-based information available at the point of care improves patient care outcomes. Online knowledge bases can increase the application of evidence-based medicine and influence patient outcome data which may be captured in quality registries. The aim of this study was to explore the effect of use of an online knowledge base on patient experiences and health care quality.

**Methods:**

The study was conducted as a retrospective, observational study of 24 primary health care centers in Sweden exploring their use of an online knowledge base. Frequency of use was compared to patient outcomes in two national quality registries. A socio-economic Care Need Index was applied to assess whether the burden of care influenced the results from those quality registries. Non-parametric statistical methods and linear regression were used.

**Results:**

Frequency of knowledge base use showed two groups: frequent and non-frequent users, with a significant use difference between the groups (*p* < 0.001). Outcome data showed significant higher values for all seven National Primary Care Patient Survey dimensions in the frequent compared to the non-frequent knowledge base users (*p* < 0.001), whereas 10 out of 11 parameters in the National Diabetes Register showed no differences between the groups (*p* > 0.05). Adjusting for Care Need Index had almost no effect on the outcomes for the groups.

**Conclusions:**

Frequent users of a national online knowledge base received higher ratings on patient experiences, but figures on health care quality in diabetes showed near to no correlation. The findings indicate that some effects may be attributed to the use of knowledge bases and requires a controlled evaluation.

## Background

The present study explored the effect of use of an online knowledge base on patient experiences and health care quality in primary health care centers in Sweden. The possible effects of knowledge base use on patient care outcomes in quality registries are scarcely reported. To the best of our knowledge, the present study is the first to examine such possible associations.

Medicine is a knowledge-intense area with a continuous need to keep up-to-date with the latest evidence and to apply it to everyday patient care. Evidence-based medicine connects the current best evidence with clinical practice [1, 2]. In order to apply evidence-based medicine in practice, knowledge is needed at the point of care [3]. Online knowledge bases may provide for these needs [4]. By capturing evidence-based medicine health care outcomes in medical quality registries, real-world evidence can be used to improve the quality of health care [5, 6].

Evidence-based medicine is “the use of the best available evidence for decision-making related to the treatment of a specific patient by applying results of systematic, reproducible, unbiased research in clinical practice” [7, 8]. Evidence-based medicine used at the point of care has been reported to mitigate risk, effectively improve patient care outcomes, and reduce cognitive overload which can lead to medical errors [1, 2, 7, 9]. A crucial step in evidence-based medicine is to translate the evidence and apply the results in clinical practice. Knowledge does not necessarily change practice and mere dissemination of scientific evidence may be insufficient to change professional behavior [10].

Online knowledge bases have been shown to increase the application of evidence-based medicine in clinical practice [11–13]. In this study, we used Lobach’s definition of a knowledge base: “Electronic (computer-based) resources comprising distilled (synthesized) or curated information that allows clinicians to select content germane to a specific patient to facilitate medical decision making” [14]. The use of knowledge bases is associated with a positive impact on clinician behavior and patient outcomes, and evidence suggests that use of knowledge bases may be associated with improved knowledge and patient outcomes [12, 15, 16].

The knowledge base explored in this study was Medibas, a web-based knowledge source for general practitioners in Sweden providing access to evidence-based medical knowledge in everyday clinical life [17]. Medibas’ editorial staff of general practitioners and its network of over 200 specialist doctors gather and summarize new scientific studies, reviews, national guidelines and recommendations and incorporate these into the knowledge base.

A national quality registry contains individual-based information on diagnoses and medical outcome measures in health care. National quality registries can identify factors that may impact on patient survival [18]. There are over a hundred national quality registries in Sweden [19]. Quality registries have the potential to collect real-world data, i.e. data collected outside of randomized controlled trials showing the unbiased results of real-life daily clinical practice. Patient experience data can be collected as patient reported outcome measures—questionnaires to record their experience of health care services. This can provide an understanding of how health care interventions impact on patients’ quality of life and allows for comparisons of health care providers’ performances [20]. Real-world data can also be collected in quality registries as objective outcome data, e.g. laboratory results or findings in physical examinations [21–24].

There is a lack of knowledge on whether the use of knowledge bases reflects the patient outcomes data in nationwide quality registries and whether burden of care, measured in Sweden as a Care Need Index influences the register outcomes in any way [25].

The aim of this study was to explore the effect of use of an online knowledge base on patient experiences and health care quality.

## Methods

### Study design and setting

A retrospective, observational ecological study design was used in the present study. Data on frequency of use of the knowledge base during 2018 was collected from primary health care centers in Stockholm, Sweden.

**Fig. 1:**
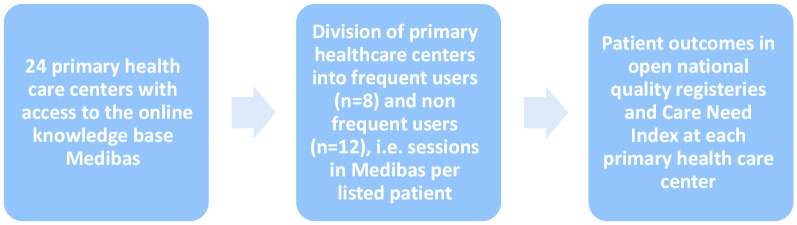
Flowchart of data sampling. The open national quality registries used were: The Swedish National Primary Care Patient Survey and The Swedish National Diabetes Register. All sampled data applied to year 2018

In parallel, a cross-sectional set of standardized outcome measures were collected from two national and regional quality registries: one containing subjective data of patient experiences in health care encounters and one containing objective data of health care outcomes from diabetes care as shown in Fig. 1.

### Study material

A total of 24 privately-owned primary health care centers participated in this study. No individual physicians were selected. Eligible participating sites were primary health care centers in Stockholm that used Medibas during full year 2018, and all centers had online access to the Internet. Primary health care centers in Stockholm were chosen because they have individual IP addresses and are thereby traceable. The knowledge base’s central customer server was used to obtain data on frequency use during 2018 for each primary health care center. A “session” in the knowledge base was defined as one occasion where the user was active on the web site.

### The knowledge base

Medibas is a Swedish medical online knowledge base with a focus on primary care [17]. The primary target audience is general practitioners, but Medibas also targets other occupational groups in primary care such as nurses and physiotherapists. The aim of the knowledge base is to provide access to evidence-based knowledge in everyday clinical practice. The knowledge base is based on the ‘Norsk Elektronisk Legehåndbok’ (Norwegian Electronic Physician Handbook) which has been used by Norwegian general practitioners since the 1990s and was adapted to Swedish health care in 2013.

The knowledge base used for this study contains more than 4000 articles and covers a wide range of diagnoses in healthcare. The texts are written by specialists in general medicine and are reviewed and adapted to Swedish guidelines. In addition to facts about symptoms, diagnostics, treatment and follow-up, Medibas also features illustrations and patient information which can be easily printed out. Each text also contains references with direct links to studies in PubMed, the Cochrane library and national or regional guidelines. The content is updated on a weekly basis to include new findings from national and international evidence-based sources of knowledge. Longer texts feature a summary at the beginning to provide an overview. Medibas receives financial support through subscription fees and does not contain advertising or commercial promotion. This knowledge base was chosen for the present study as it is the most comprehensive knowledge base accessible to primary health care centers on a nationwide scale in Sweden. It also contains patient education handouts, which may increase the willingness of patients to be compliant, according to an earlier study [26].

### The National Primary Care Patient Survey

The National Primary Care Patient Survey is a recurrent national survey of patient experiences [27]. Since 2009, all Swedish health care regions (n = 21) have participated and the survey is coordinated by the Swedish Association of Local Authorities and Regions. The survey is carried out every 2 years and includes both primary and specialized care. The most recent survey in Stockholm was carried out in 2018 (n = 57,384) and the response rate was 35.4%. Thus, 20,313 patients responded to the questionnaire. A random sample of patients who had visited primary health care centers received an invitation to respond to a web or postal questionnaire. Confidentiality was ensured and it was not possible to read an individual’s responses when the results were compiled. The questionnaire consisted of seven dimensions on a five to seven graded Likert scale: overall impression, emotional support, participation and involvement, respect and treatment, continuity and coordination, information and knowledge, and accessibility.

### The National Diabetes Register

The National Diabetes Register, founded in 1996, has long been a cornerstone of diabetes care in Sweden, providing clinicians with evidence-based information and supporting the improvement of health care quality [28]. Both hospitals and primary health care centers input diabetes patient data online, and in return benefit from opportunities to monitor risk factors, receive help in identifying needs for treatment improvements, and minimize the potential consequences of diabetes. The National Diabetes Register currently contains data on 425,000 patients and has a coverage of 97% of all Swedish diabetic patients. Data is either entered manually and reported online or directly by transmission from the patient’s electronic medical records [26]. The Swedish Society for Diabetology is the owner of the registry and receives financial aid from the Swedish Association of Local Authorities and Regions. There are over 50 variables for each patient in the National Diabetes Register, e.g. blood pressure, HbA1c and blood lipids.

### National quality registries

Data was extracted from the National Primary Care Patient Survey and the National Diabetes Register on a single occasion in April 2019 for the time period of a full 12 months from January to December 2018. Eligible data sets had no missing data. The number of sessions in the knowledge base per listed patient during 2018 were dichotomized into two groups of primary health care centers: frequent and non-frequent users of the knowledge base. We did a binary split into these two groups in order to analyze how outcomes in the National Primary Care Patient Survey and National Diabetes Register were related to frequency of use of the knowledge base.

### Care Need Index

Care Need Index is a socio-economic needs index which describes the expected risk of developing ill health based on socio-economic factors on an individual level [25, 29].

The seven variables and their relative weights are:

**Table:** 

Care Need Index variable	Relative weight (0–9)
1. Aged over 65 years and single	6.15
2. Born abroad (Eastern Europe, Asia, Africa or South America)	5.72
3. Unemployed (or in employment measure), 16–64 years	5.13
4. Single parent with children who are 17 years or younger	4.19
5. Persons, aged one year or older, who recently moved into the healthcare center’s catchment area	4.19
6. Low educational status, 25–64 years	3.97
7. Aged under 5 years	3.23

The Care Need Index is an estimate to measure the workload of Swedish general practitioners. Compensation for socio-economic weight is paid per listed patient. Based on the socio-economic weight in Care Need Index for each listed person, an index is calculated for the entire primary health care center’s patient list, which then forms the basis for the compensation. Care Need Index is not dependent on the number of visits to the primary health care center. About 60% of the Swedish population (n = 10 million) do not sort into a Care Need Index variable while 7–8% of the population have two or more variables. In order to explore whether the outcomes data from the National Primary Care Patient Survey and the National Diabetes Register were influenced by socio-economic weights, we selected Care Need Index as a measure to reveal a potential relationship.

### Statistical analysis

Descriptive statistics (mean and standard deviation) were used to describe the knowledge base user groups and register data [30]. The Mann–Whitney test was used to calculate differences between knowledge base user groups [31]. We tested dimensions of the National Primary Care Patient Survey against Care Need Index using linear regression [32]. In order to test the seven dimensions from the National Primary Care Patient Survey against Care Need Index for the dichotomized user groups, the Care Need Index data, originally showing an uneven distribution (non-parametric), was transformed to a normal distribution (parametric) using the Johnson Transformation Method [33]. Using linear regression, we tested the difference between the National Primary Care Patient Survey dimensions, National Diabetes Register parameters and Care Need Index for the dichotomized user groups. A *p *value of < 0.05 was considered significant for all statistical analyzes. The IBM SPSS statistical software version 26 was used to analyze the data [34].

### Research hypothesis

The use of Medibas, an online medical knowledge base, correlates to health care quality as measured in patient outcome data captured in national quality registries.

## Results

**Fig. 2:**
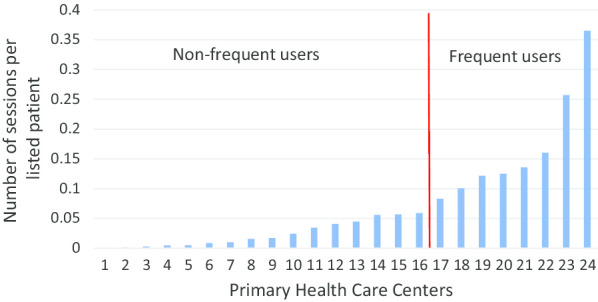
The distribution of sessions in the knowledge base per listed patient for participating primary health care centers

Frequency of use, as measured by the number of sessions in the knowledge base, and the division of the 24 primary health care centers into two groups: frequent users and non-frequent users are displayed in Fig. 2. The number of listed patients was on average 8903 in the frequent user group and 11,911 in the non-frequent user group. The listed patients were equally distributed (*p* = 0.320) between the two groups [Table 1 Care Need Index was 1.95 in the frequent user group and 2.55 in the non-frequent user group (*p* = 0.052)].

**Table 1:** Characteristics of the two user groups of the knowledge base, presented as mean values, standard deviations

Variables	Frequent users (n = 8) (SD)	Non-frequent users (n = 16) (SD)	*P* value^a^
Listed patients at the primary health care centers	8903 (2769)	11,911 (6067)	0.320
Care Need Index	1.95 (0.40)	2.55 (0.86)	0.052
Sessions per 1000 listed patient	169 (95.39)	24 (21.48)	0.000*
Response rate as percent of National Primary Care Patient Survey	39.91 (4.21)	33.53 (1.55)	0.013*
Mean age in National Diabetes Register	68.62 (0.68)	67.20 (0.66)	0.548

^a^Mann–Whitney test, exact significance 2-tailed

The frequency of sessions per listed patient was significantly higher in the frequent user group than in the non-frequent user group (*p* < 0.001), as was the response rate for the National Primary Care Patient Survey, 39.91% vs. 33.53% (*p* = 0.013) (Table 1).

### Patient experiences

All seven dimensions of the National Primary Care Patient Survey—overall impression, emotional support, participation and involvement, respect and treatment, continuity and coordination, information and knowledge, and accessibility—were rated significantly higher among patients listed at primary health care centers in the frequent user group vs. those in the non-frequent user group (Table 2).

**Table 2:** Results from the National Primary Care Patient Survey for the frequent and non-frequent user groups, mean values and (standard deviations)

Dimensions in the national primary care patient survey	Frequent user group (n = 8) (%) (SD)	Non-frequent user group (n = 16) (%) (SD)	*P* value^a^
Overall impression	89.05 (3.67)	77.12 (8.30)	0.001*
Emotional support	84.25 (4.44)	72.11 (9.24)	0.001*
Participation and involvement	87.14 (2.77)	77.52 (5.76)	< 0.001*
Respect and treatment	91.20 (2.31)	82.14 (7.29)	0.001*
Continuity and coordination	85.90 (4.49)	72.14 (9.20)	0.001*
Information and knowledge	84.25 (3.82)	72.84 (6.76)	< 9.001*
Accessibility	88.45 (2.94)	80.58 (5.97)	0.001*

^a^Mann–Whitney test, exact significance 2-tailed

### Health care quality

The National Diabetes Register parameters encompass different criteria, from medical history and physical findings such as blood pressure measurements and foot examinations to laboratory test results of blood and urine. There was no significant difference in the National Diabetes Register parameters between the user groups except for the parameter “HbA1C > 70”, which was significant (*p* = 0.045) and therefore separated the user groups (Table 3).

**Table 3:** Results for the the National Diabetes Register parameters used in the study for frequent and non-frequent user groups, mean values and (standard deviations)

The National Diabetes Register parameters	Frequent users (n = 8) (%) (SD)	Non-frequent users (n = 16) (%) (SD)	*P* value^a^
HbA1c < 52 mmol/mol	61.05 (7.64)	58.44 (4.76)	0.365
HbA1c > 70 mmol/mol	7.01 (1.83)	9.05 (2.27)	0.045*
Blood pressure ≤ 130/80 mm Hg	39.86 (6.71)	42.79 (5.17)	0.097
Blood pressure < 140/85 mm Hg	57.21 (9.13)	56.07 (5.18)	0.912
LDL < 2.5 mm/L	51.91 (7.03)	51.18 (8.36)	0.765
Lipid lowering drug	44.06 (12.35)	52.26 (9.38)	0.115
Albuminuria	24.44 (5.46)	23.01 (4.97)	0.717
Foot exam	76.83 (8.01)	81.55 (12.58)	0.119
Retinal examination	77.40 (13.55)	75.78 (26.06)	0.265
Smoker	12.63 (4.27)	15.30 (3.50)	0.184
Physically inactive	14.78 (8.43)	22.57 (8.90)	0.065

^a^Mann–Whitney test, exact significance 2-tailed

### Adjusting for Care Need Index

In order to adjust for the possible effect of Care Need Index, an additional multiple regression analysis was performed (not shown). The addition of Care Need Index of the primary health care centers in a multiple regression analysis did not affect the statistical outcomes from the National Primary Care Patient Survey. For outcomes from the National Diabetes Register, the addition of Care Need Index of the primary health care centers changed the parameter “HbA1C > 70” from significant to non-significant (*p* > 0.05) and the parameter “Physical inactive” from non-significant to significant (*p* < 0.05), i.e. significantly higher percentage physical inactive patients reported to the National Diabetes Register among primary health care centers with non-frequent use of the knowledge base. Adding mean age of primary health care centers’ patients reported to the National Diabetes Register did not affect the results from the multiple regression analysis.

## Discussion

Primary health care centers using the knowledge base grouped into two categories: frequent and non-frequent user groups. The number of sessions in the knowledge base per listed patient, i.e. frequency of use of Medibas, showed a significant difference between these two groups.

The results showed that primary health care centers using the knowledge base frequently scored higher in the National Primary Care Patient Survey. This may suggest that physicians who use the knowledge base frequently are able to fulfil the needs of patients more effectively. The National Primary Care Patient Survey contains subjective ratings by patients and reflects their views on the standard of care. The results from the National Diabetes Register showed no differences between frequent and non-frequent users of the knowledge base. This could indicate that diabetic variables such as average blood glucose level, blood lipids and blood pressure are more static and the features influencing these values are multifactorial, genotype- and phenotype-wise. Physicians’ use of electronic knowledge bases might have a diminutive influence on these physical parameters. Other researchers have found that some behavioral changes can be more easily moderated than physical parameters [35]. Based on the findings of the present study, there may be a relationship between patient outcome measurements in the National Primary Care Patient Survey and frequency of use of the knowledge base. On the other hand, objective parameters such as those in the National Diabetes Register may not be impacted by the use of an online knowledge base. It cannot be ruled out that there is no effect but results of this study neither confirm nor refute this.

Care Need Index describes the expected risk of developing ill health based on socio-economic factors and could therefore possibly affect how burden of care influences patient outcomes between frequent and non-frequent users of the knowledge base. It could be argued that patients with low expected risk of developing ill health due to socio-economic factors tend to cluster in primary health care centers where physicians are highly committed to satisfying individual patients’ needs and demands. We therefore added Care Need Index to test this hypothesis. We found no effect (apart from one minor parameter in the National Diabetes Register) when adding Care Need Index to the two groups. This may reflect that burden of care is already embodied in the National Primary Care Patient Survey as well as in the results from the National Diabetes Register. The addition of Care Need Index, whose inherent values may already be reflected in the register data, seems to have small effect and therefore not susceptible to influence from the knowledge provided by the knowledge base.

Previous research has focused on interventions to either increase the use of, or find new ways of using, electronic knowledge sources, whereas this study specifically examined the effect of the use of a knowledge base on patient outcome measures in two nationwide registries. Earlier studies have found that use of register data may play a vital role in patient care [12, 15, 36]. Furthermore, an excess of research has been devoted to evaluating electronic knowledge sources by employing self-reported use, which is prone to biases [37–39]. We are not aware of any prior studies examining outcomes of the use of an online knowledge base by relating frequency of use to objective data from quality registries. Recent studies have given valuable clarification on factors influencing knowledge seeking such as lack of time, resource use and accessibility [4, 40–42]. The present study adds to these findings by exploring the frequency of use of an online knowledge base and investigating its potential impact on unbiased outcome measures, e.g. objective outcome data from quality registries. The study brings new knowledge of how to evaluate the use of a clinical knowledge base and its possible impact on health care quality. The technical novelty in this study lies in its front line approach to evaluating the effects of clinical knowledge applied to patient care. To the best of our knowledge there are few, if any, studies which take this new approach to evaluation. Previous studies in this field have mostly been in the form of self-assessments, and thereby subject to recall bias, whereas our study looks at objective data of knowledge base use combined with results from national quality registries [33–35].

The National Primary Care Patient Survey was chosen for the study as it represents a high-quality nationwide patient reported outcomes program. The National Diabetes Register was chosen because it is the quality register for diabetes care in Sweden and contains nationwide diabetes data of high validity, reliability and granularity. The collected information is objective as it is transferred automatically from the electronic patient records.

### Strengths and limitations

This study suffers from several limitations including major confounding issues. The study design only looks at associations and not evidence for causation. Hence the conclusions of how a knowledge base can improve health care quality has to be interpreted with great care.

The purpose of this “first of its kind” study was to establish a starting point for a non-self-reported way of looking at collected user data. In the future there needs to be a focus on individual data, both from the user perspective as well as from the patient’s side.

Measuring effects of health care by studying outcomes data, e.g. in quality registries, has inherent limitations and should primarily be used for hypotheses generation. Other limitations in this study are the small sample size of the study population, the response rates of patient surveys, the low frequency of response in the National Primary Care Patient Survey and the selection of privately-owned primary health care centers. The National Primary Care Patient Survey represents an important qualitative source of information on patient preferences, but it may be difficult to extrapolate our findings to other contexts. The generalizability of our findings into other areas, such as rural geographical areas and publicly-run primary health care centers, may be limited. Further, the use of “sessions” in the knowledge base may not adequately reflect actual use of the knowledge base. Meanwhile, strengths of this study include the use of objective outcome measures (e.g. quality register data) and no self-reported results. Another strength is that a total geographical group of the knowledge base’s users was investigated. Finally, the frequent and non-frequent user groups of the knowledge base have significant differences. We believe that these differences are characteristics of the two groups; the frequent users tend to have more satisfied patients who consequently score higher on satisfaction of given care. Vice versa does the non-frequent user group seem to have less satisfied patients.

Future research in this field is needed in the form of results from an unbiased selection of patients’ and caregivers’ experiences of knowledge base use in the form of a randomized controlled trial. It should aim to find new methods to support causation between the use of a knowledge base and impact on health care quality.

## Conclusions

Frequent users of a national online knowledge base received higher ratings on patient experiences, but figures on health care quality in diabetes showed near to no correlation.

The findings indicate that some effects may be attributed to the use of knowledge bases and requires a controlled evaluation.

## Data Availability

The data sets in the present study can be made available from the corresponding author on request.

## Abbreviations

HbA1c: Hemoglobin that is chemically linked to a sugar
IBM: International Business Machines Corporation
LDL: Low-density lipoprotein
SD: Standard deviation
SPSS: Statistical package for the social sciences
